# Has the COVID-19 Pandemic Affected Breast Cancer Stage and Surgical Volume?

**DOI:** 10.3389/fsurg.2022.811108

**Published:** 2022-02-07

**Authors:** Halil Kara, Akif Enes Arikan, Onur Dulgeroglu, Burcin Tutar, Fatma Tokat, Cihan Uras

**Affiliations:** ^1^Vocational School of Health Sciences, Acibadem Mehmet Ali Aydinlar University, Istanbul, Turkey; ^2^Department of General Senology, Research Institute of Senology, Acibadem Mehmet Ali Aydinlar University, Istanbul, Turkey; ^3^Department of General Surgery, School of Medicine, Acibadem Mehmet Ali Aydinlar University, Istanbul, Turkey; ^4^Department of Radiology, Acibadem Maslak Hospital, Istanbul, Turkey; ^5^Department of Pathology, School of Medicine, Acibadem Mehmet Ali Aydinlar University, Istanbul, Turkey

**Keywords:** breast, cancer, cancer staging, breast surgery, COVID-19

## Abstract

**Background:**

This study investigates the effects of COVID-19 on the breast cancer stage and the volume of breast cancer surgery in a specialized breast institute.

**Methods:**

Data of 332 patients who were diagnosed and treated for breast cancer between December 2019 and November 2020 were evaluated retrospectively according to periods of pandemic.

**Results:**

A significant decrease in the number of operations, especially upfront surgeries rather than surgeries after neoadjuvant chemotherapy, was detected in the early period of the COVID-19 pandemic. It was found that patients with complaints were mostly admitted during this period (*p* = 0.024). No statistical significance was found for age, sex, side of the tumor, type of tumor, surgery to breast, and axilla. Following the early period of the pandemic, it was observed that patients with mostly luminal, early-stage, and less axillary nodal involvement (*p* < 0.05) were admitted, and as a result, it was founded that upfront surgeries increased, although no change in TNM staging was observed. However, it did affect the decision of initial treatment. Thus, the number of upfront surgeries was significantly higher than the NCT group (*p* = 0.027) following the early period.

**Conclusion:**

Surgical volume is significantly affected in the early period of the COVID-19 pandemic. To overcome overload due to delayed surgeries related to pandemics, some hospitals should be spared for oncological treatments. Following the early period, mostly luminal type, early-stage patients were admitted, probably because of increased self-awareness and short wave duration, but the breast cancer stage was not affected.

## Introduction

The coronavirus-19 (COVID-19) outbreak, which started in Wuhan, China, in December 2019, rapidly spread to the whole world (1, 2). On March 11, 2020, the World Health Organization reported that it was a global health problem and declared as a pandemic (1, 2). Due to the rapid spread of the pandemic, conservation of hospital beds for COVID-19 patients, and the introduction of lock-down measures, hospital admissions and screening programs for reasons other than COVID-19 had come to a halt in many countries, however, there were variation between countries in term of cancer management (3, 4).

The first case was reported in Turkey on March 11, 2020 (1, 2). With the increase of cases, both state and private hospitals were declared as pandemic hospitals and a circular was issued by the Ministry of Health of Turkey to stop surgeries other than emergency and cancer surgeries (2). In Turkey, admissions to hospitals for reasons other than COVID-19 also decreased. This was because of the rapid spread of the pandemic, the declaration of hospitals as pandemic hospitals, emphasizing the importance of social isolation and encouraging social isolation, and curfews including those people over the age of 65 years and with comorbidities.

The number of operations in surgical clinics was affected due to the postponement of elective operations during the pandemic period and the COVID-19 anxiety of patients. The decrease in the number of operations foreshadows a serious increase in the workload on surgical clinics in the post-pandemic period. Also, there is concern that the pandemic may cause delays in diagnosis, especially in tumors of general interest such as breast cancer, and therefore, result in more advanced stage tumors and disease progression.

This study investigates the effects of the COVID-19 pandemic on the breast cancer stage and the volume of breast cancer surgery in a specialized breast institute.

## Materials and Methods

Data of the patients who were diagnosed with breast cancer and had an operation or received neoadjuvant chemotherapy in Acibadem University Research Institute of Senology (RISA) affiliated two hospitals (with 300 patient beds in each) were retrospectively analyzed. The study was carried out within RISA.

Patients who underwent surgery between December 2019 and November 2020 were evaluated retrospectively to assess the surgical volume. Patients were divided into two groups as the upfront surgery group (surgery group) and the group that underwent surgery after neoadjuvant chemotherapy (NCT group), then the monthly number of operations were compared.

Separately, to examine the effects of COVID-19 on breast cancer; patients were divided into four groups (depending on COVID-19 data of Republic of Turkey Ministry of Health) according to the time of diagnosis (5):

1. Pre-pandemic period: December 2019–March 2020 as a control group.
2. First wave: April and May 2020, when the impact of the pandemic in Turkey was seen, as well as all hospitals being declared pandemic hospitals.
3. Inter-pandemic period: June–September 2020, following the first wave, the number of COVID-19 patients decreased, lockdown measures relaxed, elective surgeries and clinic services started again.
4. Second wave: October–November 2020, when the number of COVID-19 patients again increased. During this period, 25% capacity of hospitals was reserved for COVID-19 patients, but elective surgeries and outpatient services were ongoing.

The number of patients, age, gender, tumor side, presence, or absence of symptoms (mass, discharge, retraction, pain etc), types of surgery, tumor size, axillary lymph node status, stage at the time of admission, tumor biology, and type of initial treatment (surgery or neoadjuvant chemotherapy) were evaluated according to these groups.

The date of diagnosis was recorded according to needle biopsy. For staging of breast cancer, pTNM was used for the surgery group, whereas cTNM was used for the NCT group according to American Joint Committee on Cancer 8th Edition TNM classification (6). Immunohistochemical biomarkers were obtained from the postoperative pathology in the surgery group and from the core-biopsy in the NCT group. The patients were divided into three groups according to their immunohistochemistry, as luminal group, Her2 (Human Epidermal Growth Factor Receptor 2) group, and triple-negative group (6).

The operations were classified as breast-conserving surgeries (BCS) and mastectomies. While BCS included surgeries in which the entire breast was not removed, the mastectomy group included simple, skin-sparing, and nipple-sparing mastectomies. Axillary surgeries were examined in two groups as sentinel lymph node biopsy and axillary lymph node dissection.

### Statistical Analysis

Study data were collected and managed using REDCap (Research Electronic Data Capture) electronic data capture tools hosted at Acibadem Mehmet Ali Aydinlar University (7). All statistical analyses were performed using SPSS (IBM SPSS Statistics for Windows, Version 25.0. Armonk, NY: IBM Corp.). A *p* < 0.05 was considered statistically significant. Student *t*-test and ANOVA were used for continuous variables, and chi-square or Fisher's exact test was used for categorical variables. Continuous data are reported as the mean ± standard deviation.

## Results

The data of 332 patients who were diagnosed with breast cancer, underwent upfront surgery or started neoadjuvant chemotherapy between December 2019-November 2020 were retrospectively evaluated.

**Figure 1:**
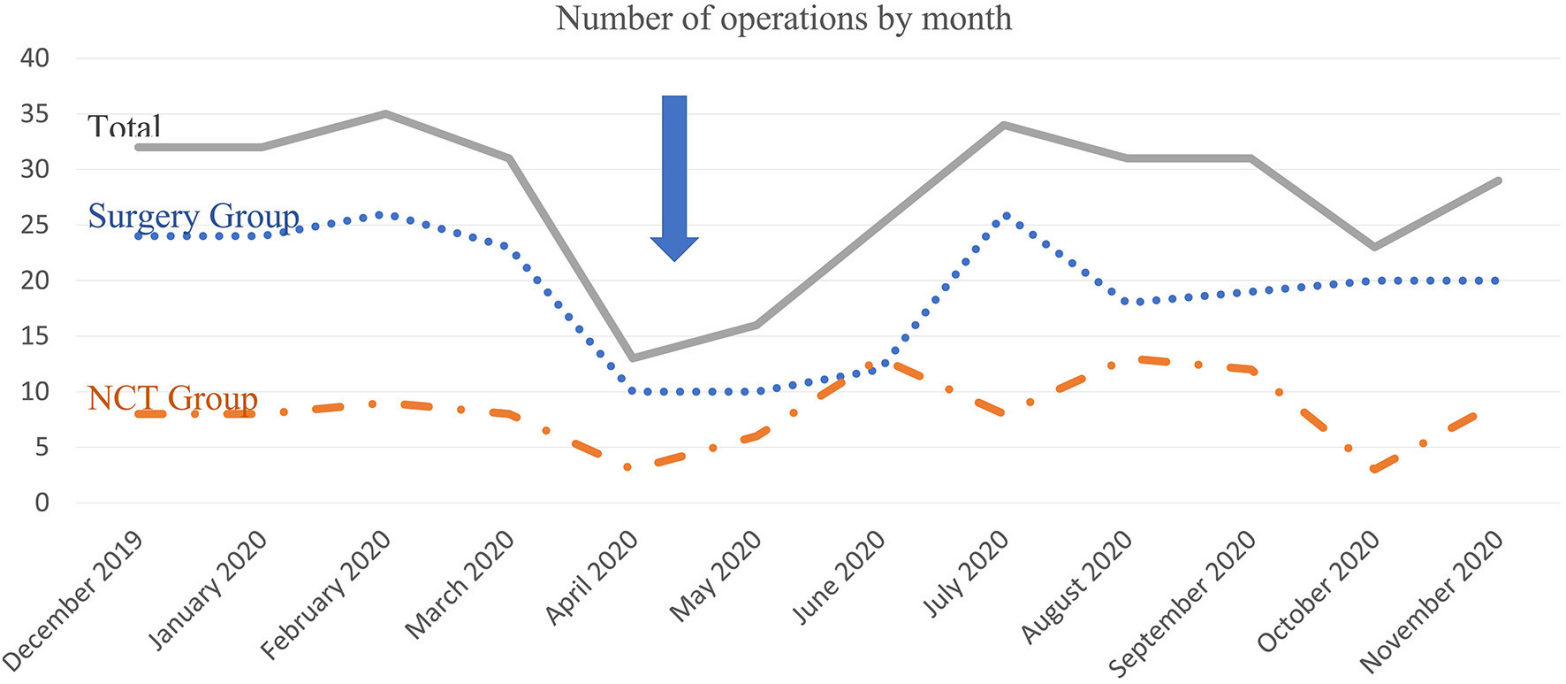
Number of operations by month.

The number of surgeries for breast cancer decreased significantly in the first wave of the pandemic. In total, this decrease is about 50–60% in the first wave compared to the pre-pandemic period. During the inter-pandemic period, the number of operations increased and approached a similar level at the pre-pandemic period. If it is considered that, breast cancer treatment was carried on as in pre-pandemic period in the institute according to decision of multidisciplinary team meeting, the decrease in upfront surgeries performed due to early-stage breast cancer is the reason for this change in the first wave rather than locally advanced breast cancer. The number of operations by month is shown in Figure 1.

Based on the date of diagnosis, the patients' data are shown in Table 1. No statistical significance was found for age, sex, side of the tumor, type of tumor (*in situ* or invasive), surgery to breast, and axilla in the analysis performed between pre-pandemic, first wave, inter-pandemic, and second-wave periods (*p* > 0.05). Considering whether the patients had symptoms at the time of admission or not, a statistical difference was detected between the groups, and it is found that patients with symptoms mostly admitted to the hospital in the first wave (*p* < 0.05). However, the statistical significance disappears after the first wave.

**Table 1:** Characteristics of the patients according to the groups.

		**Prepandemic**	**First wave**	**Interpandemic**	**Second wave**	***P*-value**
Number of cases		140	31	100	45	
Age (years)		51.3 ± 12.0	50.3 ± 11.2	51.2 ± 12.7	51.0 ± 13.3	0.888
Sex	Female	138 (98%)	31 (100%)	98 (98%)	44 (98%)	0.859
	Male	2 (2%)	0 (0%)	2 (2%)	1 (2%)	
Side of tumor	Left	71 (50%)	17 (55%)	54 (54%)	21 (47%)	0.932
	Right	69 (50%)	14 (45%)	46 (46%)	24 (53%)	
Symptom	Present	95 (68%)	28 (90%)	76 (76%)	29 (64%)	0.024*
	Absent	45 (32%)	3 (10%)	24 (24%)	16 (36%)	
Type of tumor	*in situ*	10 (7%)	3 (10%)	9 (9%)	8 (18%)	0.112
	Invasive	130 (93%)	28 (90%)	91 (91%)	37 (82%)	
Primary treatment (all cases)	Surgery	98 (70%)	17 (55%)	79 (79%)	38 (84%)	0.027*
	NCT	42 (30%)	14 (45%)	21 (21%)	7 (16%)	
Primary treatment (invasive cases)	Surgery	88 (68%)	14 (50%)	70 (77%)	38 (84%)	0.041*
	NCT	42 (32%)	14 (50%)	21 (23%)	7 (16%)	
Surgery to breast (all cases)	BCS	83 (60%)	19 (61%)	49 (52%)	19 (50%)	0.653
	Mastectomy	56 (40%)	12 (39%)	46 (48%)	19 (50%)	
Surgery to breast (invasive cases)	BCS	77 (60%)	16 (57%)	44 (51%)	17 (57%)	0.653
	Mastectomy	52 (40%)	12 (43%)	42 (49%)	13 (43%)	
Surgery to axilla (all cases)	SLNB	93 (68%)	19 (70%)	75 (80%)	30 (77%)	0.234
	ALND	44 (32%)	8 (30%)	18 (20%)	9 (23%)	
Surgery to axilla (invasive cases)	SLNB	84 (66%)	19 (73%)	67 (79%)	22 (71%)	0.234
	ALND	44 (34%)	7 (27%)	18 (21%)	9 (29%)	
Nodal status (all cases)	Negative	84 (60%)	14 (45%)	73 (73%)	30 (67%)	0.045*
	Positive	56 (40%)	17 (55%)	27 (27%)	15 (33%)	
Nodal status (invasive cases)	Negative	74 (57%)	11 (39%)	64 (70%)	22 (60%)	0.049*
	Positive	56 (43%)	17 (61%)	27 (30%)	15 (40%)	
Tumor size (mm)	24.6 ± 21.7	25.7 ± 20.5	27.0 ± 24.0	31.9 ± 25.7	0.361
T in invasive cancers	1	70 (54%)	16 (57%)	48 (53%)	18 (49%)	0.646
	2	54 (41%)	11 (40%)	38 (42%)	18 (49%)	
	3	5 (4%)	0 (0%)	4 (4%)	1 (2%)	
	4	1 (1%)	1 (3%)	1 (1%)	0 (0%)	
N in invasive cancers	0	74 (67%)	11 (39%)	64 (70%)	22 (59%)	0.093
	1	48 (37%)	15 (54%)	18 (20%)	11 (30%)	
	2	3 (2%)	1 (3%)	4 (4%)	1 (3%)	
	3	5 (4%)	1 (3%)	5 (6%)	3 (8%)	
TNM stage in invasive cancers	1	54 (42%)	8 (28%)	38 (42%)	14 (38%)	0.624
	2	65 (50%)	17 (61%)	40 (44%)	18 (49%)	
	3	11 (8%)	3 (11%)	13 (14%)	5 (13%)	
Biologic subtype	Luminal	88 (68%)	16 (57%)	70 (77%)	34 (92%)	0.038*
	Her2	30 (23%)	9 (32%)	14 (15%)	3 (8%)	
	TN	12 (9%)	3 (11%)	7 (8%)	0 (0%)	

NCT, neoadjuvant chemotherapy; BCS, breast conserving surgery; SLNB, sentinel lymph node biopsy; ALND, axillary lymph node dissection; Her2, Human Epidermal Growth Factor Receptor 2; TN, triple negative. *statistically significant.

In the comparison between the groups, no statistical difference was found in terms of tumor size for both invasive and non-invasive tumors. Nodal involvement was evaluated only for invasive cancers; while there was no difference between pre-pandemic and first wave, significantly less nodal involvement was detected in the inter-pandemic and second wave (*p* < 0.05). Therefore, although there was a statistically significant increase in the number of N0 patients in inter-pandemic and in the second wave (*p* < 0.05), this did not create a statistical significance in TNM staging (*p* > 0.05), however, this did affect the decision to start with upfront surgery or neoadjuvant chemotherapy, and the primary surgical group was significantly higher than the NCT group in invasive tumors during inter-pandemic and the second wave. When the biological subtypes were examined, the luminal group was statistically more in the second wave compared to both the pre-pandemic and first wave periods (*p* = 0.011 and *p* = 0.003, respectively).

## Discussion

In the current study, a significant decrease in the number of patients treated with upfront surgery was detected in the early period of the COVID-19 pandemic. It was found that patients with complaints were mostly admitted during this period. Following the early period of the pandemic, it was observed that patients with mostly luminal, early-stage, and less axillary nodal involvement were admitted, and as a result, it was observed that upfront surgery patients increased, but this did not cause any change in TNM staging.

Since December 2019, the COVID-19 pandemic has affected the whole world (1). Due to the rapid spread, many countries have taken lock-down measures. Many suggestions have been published for the management of breast cancer in the early period of the COVID-19 pandemic. For reasons such as directing hospital resources to COVID-19 patients, protecting healthcare personnel, high risk of postoperative pulmonary complications, and mortality in COVID-19 positive patients, there were opinions that the surgeries should be postponed until the conditions improve and become suitable for the surgery (8–11).

However, there are studies reporting that safe surgery can be performed under appropriate conditions during the pandemic period (12–19, 34). In the COVIDSurg group's study involving 9,171 patients, postoperative COVID-19 infection rate and pulmonary complication rates were lower in the COVID-19-free surgical pathway (complete segregation of the operating theater, critical care, and inpatient ward areas), and they recommended that dedicated COVID-19-free surgical pathways should be established to provide safe elective cancer surgery during current and future COVID-19 outbreaks (17). In the centers where the study was conducted, surgeries could be carried on with required precautions due to existence of covid-free surgical pathways.

Reasons such as the rapid spread of the COVID-19 pandemic, the declaration of all hospitals as pandemic hospitals during the first wave, emphasizing the importance of social isolation and encouraging social isolation, curfews that cover people over the age of 65 years and with comorbidity affected the number of patients. Combined with the anxiety of COVID-19, there has been a delay in the diagnosis of breast cancer, as the admissions of both patients with complaints and patients with routine screening have decreased (20–22). This situation has significantly affected surgical clinics. The number of operations dropped in the first wave of the pandemic (13, 23, 24). Li et al. (23) reported that new breast cancer diagnoses and breast cancer surgeries have decreased. But considering the overall low infection rates, they conclude that most cancer patients, especially in low infection risk regions, should receive their regular therapy without interference. In the study of Acea-Nebril et al. (13), surgical activity decreased by 30% when patients who underwent surgery between March 16 and May 31, 2020, were compared with those who had surgery on the same time period in 2019.

In a study in which the National Health Service data was analyzed, the total referrals to breast centers decreased by 28% in the first 6 months of 2020 compared to the first 6 months of 2019 (*N* = 231,765 vs. *N* = 322,994), and this decrease was more pronounced in April and May (25). In addition, the number of patients who received their first treatment for breast cancer decreased by 16% in total during the same periods. This decrease also started in April but continued in May and June. However, since the rate of cancer diagnosis in non-urgent references is low (at 1.4%) and most of the decline in the reference is non-urgent, these authors believe that the overall decrease in the referrals will have less impact on the total cancer diagnosis. In another study, an overall decrease in the number of patients with breast cancer who were undergoing surgery in the early period of the pandemic (9 March−17 May 2020) was reported (26). As in the current study, the most significant decline was seen in the surgery for T1-T2 and N0 tumors.

It should be considered that another effect of the decrease in the number of patients during the pandemic period can be seen in the post-pandemic periods. In the study conducted by the COVIDSurg group using a Bayesian β-regression model, it was reported that a 12-week worldwide cancellation would cause the cancellation or delay of ~28,404,603 surgeries, and even if the normal surgical volume increases by 20% after the pandemic, it will take 45 weeks to clear these backlog of operations (27). The decrease in the number of patients in the current study was noticeable, especially from April to May 2020. With the attenuation of the first wave of the pandemic, the number of operations again increased. During the inter-pandemic period and the second wave, there was no decrease as in the first wave. Although the decrease in the number of surgeries in our clinic in the early period supports the prediction of the COVIDSurg group, the increase in surgeries later indicates that the expected surgical overload after the pandemic will not be by that much (27).

As seen in the current study, especially upfront surgery performed due to early-stage breast cancer has decreased in the early period of the pandemic. There was no such decrease in the inter-pandemic period and in the second wave. We consider the reason for this situation as the adaptation of both the patients and the clinic to the current pandemic conditions. We suggest that not every hospital should be declared as a pandemic hospital during pandemic periods. Instead, some hospitals should be spared for oncological treatments to prevent this decrease in operations and post-pandemic burden during intermittent pandemics like this or possible pandemics in the future. In this way, operations can continue, and the overload that may occur later can be reduced. In addition, both the anxiety of patients with current treatment can be reduced, and there will be no delays in the admission of patients with complaints.

Since December 2019, due to the lock-down measures taken for the COVID-19 pandemic and the reallocation of resources to COVID-19 patients, there have been problems in the diagnosis and treatment of many diseases, including breast cancer (3). These disruptions have led to changes in breast cancer treatment which brought about the concern that delays could lead to upstaging and disease progression, which could lead to poorer outcomes and decreased survival (4, 9–11, 25). Delays in diagnosis may lead to upstaging and cause patients to need more treatment. Larger surgeries, such as mastectomy and reconstruction, may be required, and the need for neoadjuvant chemotherapy may increase. This situation might lead to a decrease in the quality of life of the patient, a deterioration in the outcomes and an increase in the cost of treatment. Vanni et al. (4) estimated that with the suspension of screening programs, 8,125 patients may not be diagnosed with breast cancer in 3 months. If this period is increased to 6 months, this number may reach 16,250. Delays in diagnosis might lead to an increase in tumor size and stage and the need for more invasive surgeries and reconstructions.

With COVID-19, due to the temporary halt of cancer screening programs in the Netherlands, less than expected breast and colorectal cancer diagnoses were made in the early period of the pandemic, and this situation was more pronounced in breast cancer, especially in the 50–74 age group for whom screening was planned (3). In a national, population-based, modeling study on 32,583 breast cancer cases in January–December 2010 in the UK, they estimate a 7.9–9.6% increase in the number of deaths due to breast cancer up to year 5 after diagnosis, corresponding to between 281 and 344 additional deaths (28). Romics et al. (18) founded a higher tumor size, ER-negative and Her2 positive rate in patients who were operated on in the 8-week period after the lock-down was declared, and 64.8% of the patients were symptomatic. In the current study, although there was no difference in tumor size and biology compared to pre-pandemic in the first wave, it was observed that symptomatic patients were admitted in the early period.

In the studies of Vanni et al. (29), when 223 patients who were operated on between March 11 and May 30, 2020, were compared with 209 patients operated in the same period in 2019, waiting time, lymph node involvement, and cancer grading were found to be significantly different in univariate analysis, while waiting time for lymph node involvement was found to be significant in the multivariate analysis. However, it should be kept in mind that the patients included in this study are patients in the first 3 months of the pandemic period, and symptomatic patients who cannot admit due to extraordinary conditions or patients whose screening was delayed due to pandemic will admit in June 2020 and after, especially when the first wave of the pandemic is relieved.

Although Filipe et al. (26) reported that the number of surgeries in the early period of the pandemic decreased, they do not think that the decrease in patients coming from screening programs will have a significant effect on long-term outcomes because they believe that these patients are mostly in the early stage and that short-term delays will not increase the number of breast cancers in the later stages. There are publications showing that delays up to 60 days do not adversely affect oncological outcomes, especially in early-stage breast cancers, regardless of tumor biology (30–32). When this information is combined with the result of the current study, it can be postulated that the tumor stage is not affected in intermittent pandemics, especially if the duration of the waves is short.

In the current study, the number of patients in the first wave of the pandemic decreased. However, after the first wave, contrary to what was expected, luminal, earlier stage breast cancer patients without axillary nodal involvement were admitted instead of more advanced patients. It is possible that the reason for this situation may be increased self-awareness. Health was kept in the second place due to daily life and work stress. With lock-down, home office employees have increased, and the duration of stay at home has been prolonged. Patients consulted a doctor earlier due to the increase in awareness and the chance of women to spare time for this. Also, telemedicine opportunities may have a positive impact on early diagnosis with complaints (33).

In conclusion, the number of surgeries for breast cancer, especially upfront surgeries decreased by about 50–60% in the first wave of the pandemic. To avoid the effects of this reduction in surgeries, every hospital should not be declared as a pandemic hospital. Instead, some hospitals should be spared for oncological treatments during pandemic periods, so breast cancer surgeries can continue. Alternatively, intermittent measures for the pandemic could reduce the effect of pandemic on oncological outcome. Following the early period, mostly luminal type, early-stage patients were admitted probably because of increased self-awareness and short wave duration of the pandemic. This did not cause any change in TNM staging. However, it was effective in the decision of initial treatment. Thus, the number of upfront surgeries was significantly higher than surgeries after NCT.

### Limitations

The limitations of this study include the retrospective and small size sample, especially in the first wave of the pandemic. Study was conducted in tertiary referral centers specialized for breast cancer, thus, cancer detected by screening in the centers are low in number.

## Data Availability Statement

The datasets presented in this article are not readily available due to regulations of the Turkish government. Requests to access the datasets should be directed to halil.kara@acibadem.edu.tr.

## Ethics Statement

The studies involving human participants were reviewed and approved by Ethics Committee approval from the Ministry of Health of the Republic of Turkey was taken with file number Halil KARA-2021-02-15T16_06_54 and from the Ethics Committee of Acibadem University on 24.02.2021 with the number 2021/04. Patient consent forms were not obtained due to retrospective nature of the study. Written informed consent for participation was not required for this study in accordance with the national legislation and the institutional requirements.

## Author Contributions

HK, AEA, CU, and FT: study design. HK, AEA, BT, and OD: study concept. HK, AEA, and OD: data collection. HK and AEA: data analysis. HK, AEA, BT, and FT: writing. HK, AEA, CU, and OD: critical revising. All authors contributed to the article and approved the submitted version.

## Conflict of Interest

The authors declare that the research was conducted in the absence of any commercial or financial relationships that could be construed as a potential conflict of interest.

## Publisher's Note

All claims expressed in this article are solely those of the authors and do not necessarily represent those of their affiliated organizations, or those of the publisher, the editors and the reviewers. Any product that may be evaluated in this article, or claim that may be made by its manufacturer, is not guaranteed or endorsed by the publisher.
